# Accuracy of Urine Circulating Cathodic Antigen Test for the Diagnosis of *Schistosoma mansoni* in Preschool-Aged Children before and after Treatment

**DOI:** 10.1371/journal.pntd.0002109

**Published:** 2013-03-21

**Authors:** Jean T. Coulibaly, Yves K. N'Gbesso, Stefanie Knopp, Nicaise A. N'Guessan, Kigbafori D. Silué, Govert J. van Dam, Eliézer K. N'Goran, Jürg Utzinger

**Affiliations:** 1 Department of Epidemiology and Public Health, Swiss Tropical and Public Health Institute, Basel, Switzerland; 2 University of Basel, Basel, Switzerland; 3 Unité de Formation et de Recherche Biosciences, Université Félix Houphouët-Boigny, Abidjan, Côte d'Ivoire; 4 Centre Suisse de Recherches Scientifiques en Côte d'Ivoire, Abidjan, Côte d'Ivoire; 5 Centre de Santé Urbain d'Azaguié, Departement d'Agboville, Azaguié, Côte d'Ivoire; 6 Department of Parasitology, Leiden University Medical Centre, Leiden, The Netherlands; College of Public Health and Health Professions, United States of America

## Abstract

**Background:**

The Kato-Katz technique is widely used for the diagnosis of *Schistosoma mansoni*, but shows low sensitivity in light-intensity infections. We assessed the accuracy of a commercially available point-of-care circulating cathodic antigen (POC-CCA) cassette test for the diagnosis of *S. mansoni* in preschool-aged children before and after praziquantel administration.

**Methodology:**

A 3-week longitudinal survey with a treatment intervention was conducted in Azaguié, south Côte d'Ivoire. Overall, 242 preschoolers (age range: 2 months to 5.5 years) submitted two stool and two urine samples before praziquantel administration, and 86 individuals were followed-up posttreatment. Stool samples were examined with duplicate Kato-Katz thick smears for *S. mansoni*. Urine samples were subjected to POC-CCA cassette test for *S. mansoni*, and a filtration method for *S. haematobium* diagnosis.

**Principal Findings:**

Before treatment, the prevalence of *S. mansoni*, as determined by quadruplicate Kato-Katz, single CCA considering ‘trace’ as negative (t−), and single CCA with ‘trace’ as positive (t+), was 23.1%, 34.3% and 64.5%, respectively. Using the combined results (i.e., four Kato-Katz and duplicate CCA(t−)) as diagnostic ‘gold’ standard, the sensitivity of a single Kato-Katz, a single CCA(t−) or CCA(t+) was 28.3%, 69.7% and 89.1%, respectively. Three weeks posttreatment, the sensitivity of a single Kato-Katz, single CCA(t−) and CCA(t+) was 4.0%, 80.0% and 84.0%, respectively. The intensity of the POC-CCA test band reaction was correlated with *S. mansoni* egg burden (odds ratio = 1.2, p = 0.04).

**Conclusions/Significance:**

A single POC-CCA cassette test appears to be more sensitive than multiple Kato-Katz thick smears for the diagnosis of *S. mansoni* in preschool-aged children before and after praziquantel administration. The POC-CCA cassette test can be recommended for the rapid identification of *S. mansoni* infections before treatment. Additional studies are warranted to determine the usefulness of POC-CCA for assessing drug efficacy and monitoring the impact of control interventions.

## Author Summary

The strategy to control morbidity due to infection with the blood fluke *Schistosoma mansoni* is to regularly treat school-aged children with the drug praziquantel. Recent studies suggest that in highly endemic areas preschoolers might need to be included in such deworming campaigns. An accurate diagnosis is important to assess how many preschool-aged children need treatment, but the widely used Kato-Katz technique does not detect all infections. We assessed the accuracy of a point-of-care (POC) test that is based on the detection of the fluke's circulating cathodic antigen (CCA) in children's urine. We obtained two stool and two urine samples from 242 preschoolers in Côte d'Ivoire before and from 86 of these children after praziquantel treatment. Stool samples were examined with the Kato-Katz technique and urine samples with the POC-CCA test for *S. mansoni*. The sensitivity of one POC-CCA was much higher than a single Kato-Katz for *S. mansoni* diagnosis before (69.7% *versus* 28.3%) and after treatment (80.0% *versus* 4.0%). The POC-CCA therefore is useful for the rapid identification of *S. mansoni*-infected preschoolers who need treatment. The application of the POC-CCA test for monitoring of schistosomiasis control interventions needs further investigation.

## Introduction

Recognizing the public health impact of schistosomiasis and soil-transmitted helminthiasis, the World Health Organization (WHO) has set a minimum target for the control of morbidity due to these parasitic worm infections, urging member states to regularly treat at least 75% and up to 100%, of all school-aged children at risk of morbidity [1], [2]. As a result, many African countries have set up national plans of action for the control of schistosomiasis and soil-transmitted helminthiasis, and pursue school-based deworming campaigns [3], [4]. Experience and lessons from these programs are that they significantly reduce the prevalence and intensity of infection, and thus morbidity [5]–[8].

There is growing evidence that soil-transmitted helminths (*Ascaris lumbricoides*, hookworm, and *Trichuris trichiura*) and schistosome infections are acquired already in early childhood [9]–[14]. Hence, there is a need for effective and safe treatment of preschool-aged children, as their inclusion in preventive chemotherapy is being discussed [10], [11], [15], [16]. The intensity of infection with soil-transmitted helminths and schistosomes is age-dependent, usually showing a peak in school-aged children and adolescents [17], [18]. For schistosomiasis this might be due to cumulative and increasing water contacts of the school-aged child, combined with the maturation and increasing egg-laying capacity of schistosome worm pairs [11]. Hence, the majority of infected young children might excrete only a few eggs with their feces (for soil-transmitted helminths and *Schistosoma mansoni*) and their urine (for *S. haematobium*) [9], [11], [13], [17].

It is important to note that the Kato-Katz technique, which is widely used in endemic countries for the diagnosis of *S. mansoni* and soil-transmitted helminths, lacks sensitivity, particularly in areas of low endemicity, and for low-intensity infections (i.e., in young children or after treatment interventions) [19]–[22]. Hence, improved diagnostic methods for the accurate detection of *S. mansoni* in preschool-aged children, assessment of drug efficacy, and monitoring progress of control programs are desirable. Recent studies have shown that indirect diagnostic tests (e.g., point-of-care circulating cathodic antigen (POC-CCA)) have become valuable alternatives to direct parasitological methods for the diagnosis of *S. mansoni* [13], [23]. Note that the POC-CCA cassette test detects the presence of CCA (a schistosome glycoprotein) in host urine, after being regurgitated into the bloodstream by actively feeding worms, and successive clearance in the host's kidneys. Schistosome antigens (CCA and circulating anodic antigen (CAA)) can be detected in the serum and urine of infected individuals and their levels are sensitive and specific markers for the presence and intensity of infection [13], [23]–[26]. Circulating antigens disappear from serum and urine of schistosomiasis patients within a couple of weeks after successful treatment [24], [27]. Studies assessing a CCA urine dipstick and a POC-CCA cassette test in preschool-aged children in Uganda and Kenya, respectively, recommended these rapid tests as a useful technique for the detection of *S. mansoni* in that age group [11], [28], [29]. In our own research, conducted with school-aged children in south Côte d'Ivoire, we found that a single POC-CCA cassette test was similarly sensitive as triplicate Kato-Katz thick smears for the diagnosis of *S. mansoni* [26]. However, the physiological development and biological processes, such as absorption, distribution, metabolism, toxicity and, particularly, excretion are all age and setting dependent [30]. Moreover, the effect of geographical variations of *S. mansoni* strains on the performance of POC-CCA cassette test is poorly understood. Hence, there is a need to determine the accuracy of the POC-CCA cassette test in preschoolers from different settings as a diagnostic tool for *S. mansoni*, including its potential for drug efficacy evaluation, and monitoring of community effectiveness of control interventions.

The current study was designed to assess the accuracy of the commercially available urine POC-CCA cassette test for the diagnosis of *S. mansoni* in preschool-aged children. We designed a 3-week longitudinal study with a treatment intervention, and determined the accuracy of the POC-CCA cassette test before and after the administration of praziquantel.

## Methods

### Ethics Statement

Our study received ethical clearance from the Ministry of Health and Public Hygiene of Côte d'Ivoire (reference no. 4248/2010/MSHP/CNER). Local authorities in the study area (Azaguié, south Côte d'Ivoire) were informed about the objectives, procedures, and potential risks and benefits of the study. At study onset, a door-to-door information campaign was conducted, and all households in the area informed about the aims and procedures of the study. Written informed consent (or fingerprints of illiterate people) was obtained from parents/guardians of participating preschool-aged children.

Treatment was administered to all preschool-aged children and their mothers, irrespective of their infection status. Participating preschool-aged children were treated with crushed praziquantel tablets at a dose of 40 mg/kg and the efficacy and safety of this intervention have been described elsewhere [31]. At the end of the study, anthelmintic treatment (single 40 mg/kg oral dose of praziquantel against schistosomiasis, and single 400 mg oral dose of albendazole against soil-transmitted helminthiasis) was offered to all villagers free of charge.

### Study Area and Population

The study pursued a 3-week longitudinal design with a treatment intervention and was conducted between August and November 2011 in two villages located in the Azaguié district in south Côte d'Ivoire. The two villages, Azaguié Makouguié (geographical coordinates, 05°37′33″N latitude, 04°09′04″W longitude) and Azaguié M'Bromé (05°39′42″N, 04°08′38″W) are co-endemic for *S. mansoni* and *S. haematobium* [26], [31]. Subsistence farming is the main economic activity in both villages. Unprotected surface water bodies are frequently contacted due to the lack of tap water and other sources of clean water. Improved sanitary facilities are the exception rather than the norm. Our door-to-door census conducted in June 2011 revealed total populations of 931 people in Azaguié M'Bromé, and 783 people in Azaguié Makouguié. For the current study, emphasis is placed on preschool-aged children younger than 6 years in both villages (*n* = 367).

### Stool and Urine Collection

Using records obtained from the mid-2011 census, a list of all children aged <6 years (considered at preschool-age) was prepared and all of them were invited to participate in our study. Two cross-sectional parasitological surveys were implemented; at baseline and 3 weeks after the administration of praziquantel in order to study the epidemiology of schistosomiasis in preschool-aged children, assess the efficacy and safety of praziquantel in this age group, and determine the diagnostic accuracy of the POC-CCA casette test before and after treatment. Mothers/guardians of participating preschoolers were provided with two plastic containers labeled with unique identification numbers (IDs) at the first day of the respective survey. Mothers/guardians were instructed to collect a morning stool and urine sample of the child, each in one of two separate containers. After sample collection, the mothers were invited to submit the filled containers until noon to fieldworkers stationed at a central location (the primary school) in each village. Upon submission of the specimens, mothers were handed out a second set of two containers for stool and urine sample collection the next day.

### Laboratory Procedures

Stool and urine samples were transferred to a nearby laboratory located in the district town Azaguié and processed on the same day. For the diagnosis of *S. mansoni*, duplicate Kato-Katz thick smears were prepared from each stool sample, using 41.7 mg templates [32]. Kato-Katz thick smears were allowed to clear for at least 30 min before examination under a microscope by experienced laboratory technicians. The number of *S. mansoni* eggs was counted and recorded. Additionally, eggs of soil-transmitted helminths were counted and recorded for each species separately.

For the diagnosis of *S. haematobium*, urine samples were subjected to a filtration method, as described elsewhere [1], [12]. In brief, 10 ml of vigorously shaken urine were gently pressed through a filter mesh (30 µm; Sefar AG, Heiden, Switzerland). The filter mesh was placed on a microscope slide and a drop of Lugol's iodine solution added before quantitative examination under a microscope for *S. haematobium* eggs by experienced technicians.

For quality control, 10% of the Kato-Katz and the urine filtration slides were re-examined by a senior technician. In case of disagreement with the initial readings, the results were discussed with the concerned technicians and the slides read a third time until agreement was reached.

Urine samples were additionally subjected to a commercially available POC-CCA cassette test (batch no.: 33112; Rapid Medical Diagnostics, Pretoria, South Africa). The POC-CCA tests were performed as follows: one drop of urine was added to the well of the testing cassette. Once fully absorbed, one drop of buffer (provided with the CCA test kits) was added and the test results were read 20 min after adding the buffer. In case the control bands did not develop, the test was considered invalid and the urine sample was retested with a new POC-CCA cassette. Valid tests were scored as either negative or positive, the latter further stratified into trace, 1+, 2+, or 3+ according to the visibility of the color reaction and the manufacturer's instructions. All tests were read independently by two investigators. In case of discordant results, a third independent investigator was consulted, and the results were discussed until agreement was reached [26].

Stool and urine samples collected 3 weeks after the administration of praziquantel (single oral dose of 40 mg/kg using crushed tablets) were subjected to the same diagnostic tests as during the pretreatment cross-sectional survey.

### Statistical Analysis

Data were double entered into an Excel spreadsheet, transferred into EpiInfo version 3.2 (Centers for Disease Control and Prevention; Atlanta, United States of America), and cross-checked. In case of discrepancies, the results were traced back to the original data records. Statistical analyses were done using Stata version 10 (Stata Corp.; College Station, United States of America). Only children who had complete data records from the baseline surveys (i.e., quadruplicate Kato-Katz thick smears, two POC-CCA cassette tests, and two urine filtrations) were included in the final analysis.

Helminth species-specific fecal egg counts (FECs) as recorded by the microscopists were transformed into numbers of eggs per gram of stool (EPG), multiplying the FEC of each Kato-Katz reading by a factor 24. To assess the infection intensity of each individual, we calculated the arithmetic mean EPG value of quadruplicate Kato-Katz thick smear readings and categorized them according to thresholds given by WHO [1]. The three infection intensity classes for *S. mansoni* are (i) light (1–99 EPG); (ii) moderate (100–399 EPG); and (iii) heavy (≥400 EPG). Means were compared by Wilcoxon signed rank test and proportions by Pearson's χ^2^ test. Based on POC-CCA test scores, the infection intensity of *S. mansoni* was categorized into light (trace or 1+), moderate (2+) and heavy (3+). To investigate the infection intensity of all infected individuals, we calculated the group arithmetic mean of the individual arithmetic mean EPG values. When using the combined results of the POC-CCA tests from days 1 and 2, discordant scores were redefined to provide a single infection intensity measure, as shown in Table 1.

**Table 1:** Scoring scheme to obtain final urine POC-CCA cassette test results.

Day 1 score or *vis versa*	Day 2 score or *vis versa*	Final score*
Negative (0)	Trace	Negative (0)
Negative (0)	1+	1+
Negative (0)	2+	1+
Negative (0)	3+	2+
Trace	1+	1+
Trace	2+	1+
Trace	3+	2+
1+	2+	2+
1+	3+	2+
2+	3+	3+

* The final score was determined in case of discordance between test scores from days 1 and 2 and the final score was based on the sum of test scores from both days, divided by two.

For determining the POC-CCA test accuracy, ‘trace’ results were considered as negative in our ‘gold’ standard, due to the fact that ‘trace’ can indicate false positivity. Thus, the accuracy of the Kato-Katz and POC-CCA tests (considering trace results as negative (t−)) for the diagnosis of *S. mansoni* was determined. As diagnostic ‘gold’ standard before and after treatment we considered the combined results of quadruplicate Kato-Katz thick smears and duplicate CCA(t−), resulting in a positive case as both or either of the tests was positive (see also Midzi et al. [33]). This assumes an (almost) 100% specificity for the CCA(t−) test. Based on this ‘gold’ standard, sensitivity, specificity, and negative predictive value (NPV) were calculated. The strength of agreement between quadruplicate Kato-Katz thick smears and the POC-CCA test before treatment was assessed by kappa statistics (κ), as follows: κ = 0 indicating no agreement; κ = 0–0.2 indicating poor agreement; κ = 0.21–0.4 indicating fair agreement; κ = 0.41–0.6 indicating moderate agreement; κ = 0.61–0.8 indicating substantial agreement; and κ = 0.81–1.0 indicating almost perfect agreement [34], [35]. Differences of p<0.05 were considered as statistically significant.

A univariable logistic regression was performed to assess the association between POC-CCA cassette test results, expressed as binary outcome variable (negative/positive), and a schistosome infection, with separate models for *S. mansoni* and *S. haematobium*. Hence, egg counts from each schistosome species were utilized as explanatory variable (eggs per gram of stool (EPG) for *S. mansoni* and eggs per 10 ml of urine for *S. haematobium*).

## Results

### Study Adherence and Population Characteristics

**Figure 1:**
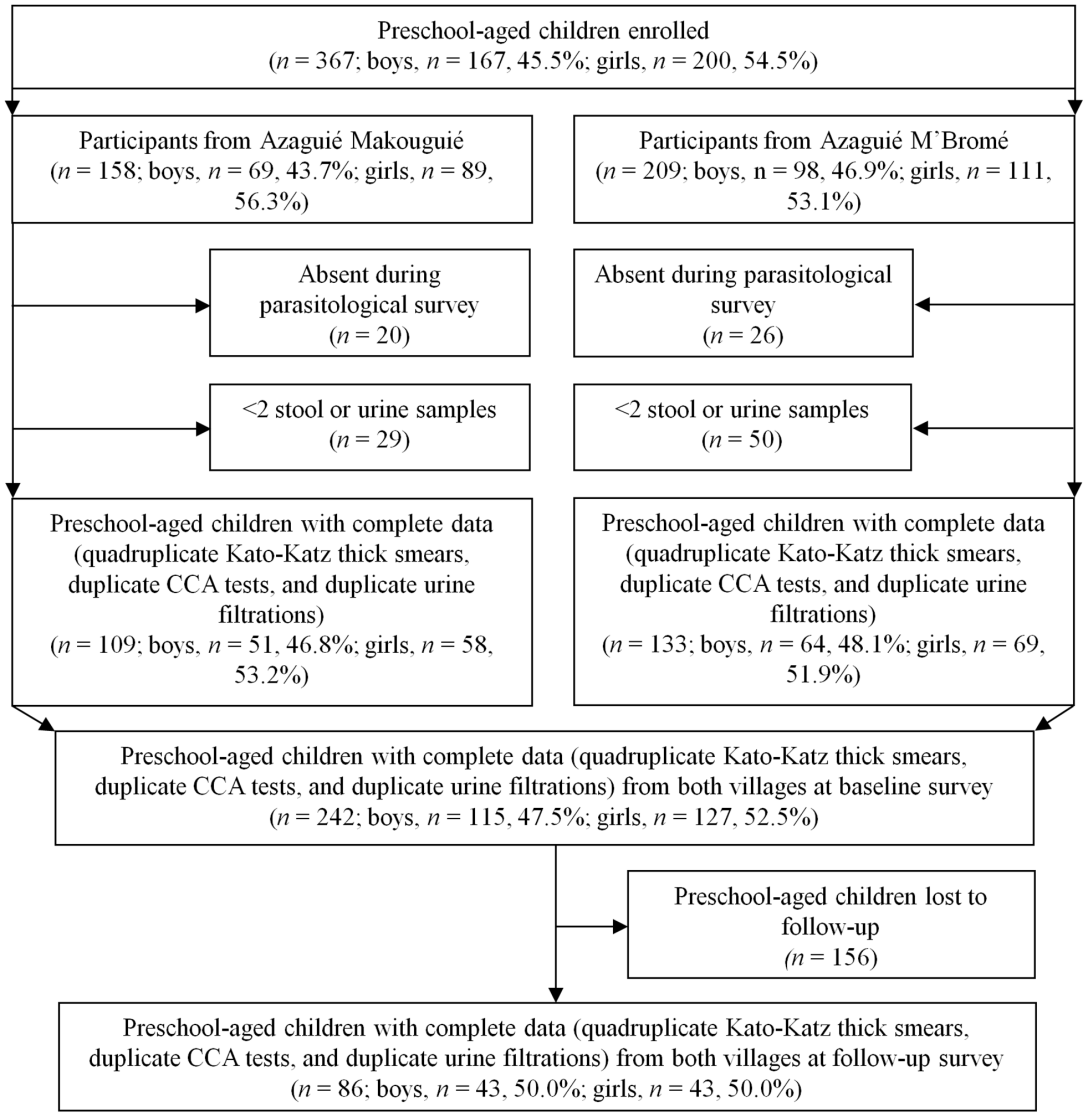
Flowchart showing study participation. Flowchart detailing study participation and adherence of preschool-aged children for submitting two stool and two urine samples for the diagnosis of *S. mansoni*, *S. haematobium*, and soil-transmitted helminths before and after administration of praziquantel in the two study villages in the Azaguié district, south Côte d'Ivoire, in August and September 2011.

Figure 1 shows that a total of 367 preschool-aged children were enrolled in the two study villages, 200 girls (54.5%) and 167 boys. Complete parasitological data (i.e., quadruplicate Kato-Katz thick smears, duplicate POC-CCA cassette tests, and duplicate urine filtrations) at the baseline survey before treatment were available for 242 children, 133 from Azaguié M'Bromé (55.0%) and 109 from Azaguié Makouguié. There were 127 girls (52.5%) and 115 boys with a mean age of 3.2 years (range: 2 months to 5.5 years).

Three weeks after the administration of praziquantel, only 86 out of the 242 children had complete parasitological data. There were 43 girls (50.0%) and 43 boys with a mean age of 3.6 years (range: 15 months to 5 years). The two population groups (children with complete data records before and after treatment) were similar in terms of average age, sex, arithmetic mean FECs of *S. mansoni*, and co-infection status (all p>0.05).

### *S. mansoni* and Soil-Transmitted Helminth Infections before Treatment

Table 2 shows the baseline prevalence and intensity of *S. mansoni* infection, as assessed by Kato-Katz and POC-CCA. Among the 242 children with complete data records, 56 (23.1%) were found positive for *S. mansoni* by quadruplicate Kato-Katz thick smears. Most infections were of light intensity (*n* = 40; 71.4%), whereas 12 children (21.4%) had a moderate (100–399 EPG) and four children (7.1%) had a heavy infection (≥400 EPG). The group arithmetic mean FEC was 23.4 EPG (95% confidence interval (CI): 13.0–33.7 EPG). A single CCA(t−) test identified 83 children (34.3%) harboring active schistosome infections.

**Table 2:** Baseline prevalence of helminths according to diagnostic approach (*n* = 242).

Parasite	Diagnostic approach	No. of infected individuals	% positive (95% CI)	Infection intensity (%)		
				Light	Moderate	Heavy
**Schistosomiasis**						
*Schistosoma mansoni*	Quadruplicate Kato-Katz thick smears	56	23.1 (17.6–28.3)	40 (71.4)	12 (21.4)	4 (7.1)
*Schistosoma mansoni* (t−)	Single POC-CCA cassette test (day 1)	83	34.3 (28.3–40.3)	40 (16.5)	14 (5.8)	29 (11.9)
*Schistosoma mansoni* (t+)	Single POC-CCA cassette test (day 1)	156	64.5 (58.4–70.5)	113 (46.7)	14 (5.8)	29 (11.9)
*Schistosoma haematobium*	Two urine filtrations	26	10.7 (6.8–14.7)	25 (96.2)	n.d.	1 (3.8)
**Soil-transmitted helminths**						
*Trichuris trichiura*	Quadruplicate Kato-Katz thick smears	22	9.1 (1.9–12.7)	22 (100)	0	0
Hookworm	Quadruplicate Kato-Katz thick smears	15	6.2 (3.1–9.3)	15 (100)	0	0
*Ascaris lumbricoides*	Quadruplicate Kato-Katz thick smears	9	3.7 (1.3–6.1)	6 (66.7)	3 (33.3)	0

The study was carried out in Azaguié, south Côte d'Ivoire in August and September 2011. Duplicate Kato-Katz thick smears were prepared from each stool sample and a single POC-CCA cassette test was done on urine samples collected over two consecutive days. Infection intensities are based on thresholds put forth by WHO [1]. The POC-CCA test results were categorized as light (1+), moderate (2+), and heavy (3+).

CI, confidence interval; n.d., not defined; POC-CCA, point-of-care circulating cathodic antigen; t−, trace negative; t+, trace positive.

The youngest child infected with *S. mansoni*, as determined by the presence of *S. mansoni* eggs in stool using the Kato-Katz technique, was 8 months. According to the CCA(t−) test results, the earliest infection was observed in a child aged 3 months.

According to quadruplicate Kato-Katz thick smears before treatment, among the 242 preschool-aged children with complete data records, 22 (9.1%), 15 (6.2%) and nine (3.7%) were positive for *T. trichiura*, hookworm and *A. lumbricoides*, respectively (Table 2). Hookworm and *T. trichiura* infections were exclusively of light intensity (<2,000 EPG and <1,000 EPG, respectively), whereas a third of the *A. lumbricoides* infections were of moderate intensity (5,000–49,999 EPG).

Among the 40 children who were infected with *S. mansoni* according to a single CCA(t−) test, but showed no *S. mansoni* eggs in any of the four Kato-Katz thick smears, three (7.5%), two (5.0%), and two (5.0%) children were positive for *T. trichiura*, *S. haematobium* and *A. lumbricoides*, respectively. None of these children were infected with hookworm.

### *S. haematobium* Infections before Treatment

Among 242 children at the baseline survey, 26 were infected with *S. haematobium*, giving a prevalence of 10.7% (Table 2). Only one child, a 5-year-old girl, had a heavy infection (128 eggs/10 ml of urine). There was no significant association between CCA(t−) results expressed as binary variable (presence/absence of disease) and *S. haematobium* egg counts (OR = 1.2; p = 0.81). Similarly, no significant association was found between CCA(t+) results expressed as binary variable (presence/absence of disease) and *S. haematobium* egg counts (OR = 1.2; p = 0.11).

### Diagnostic Accuracy before Treatment

**Figure 2:**
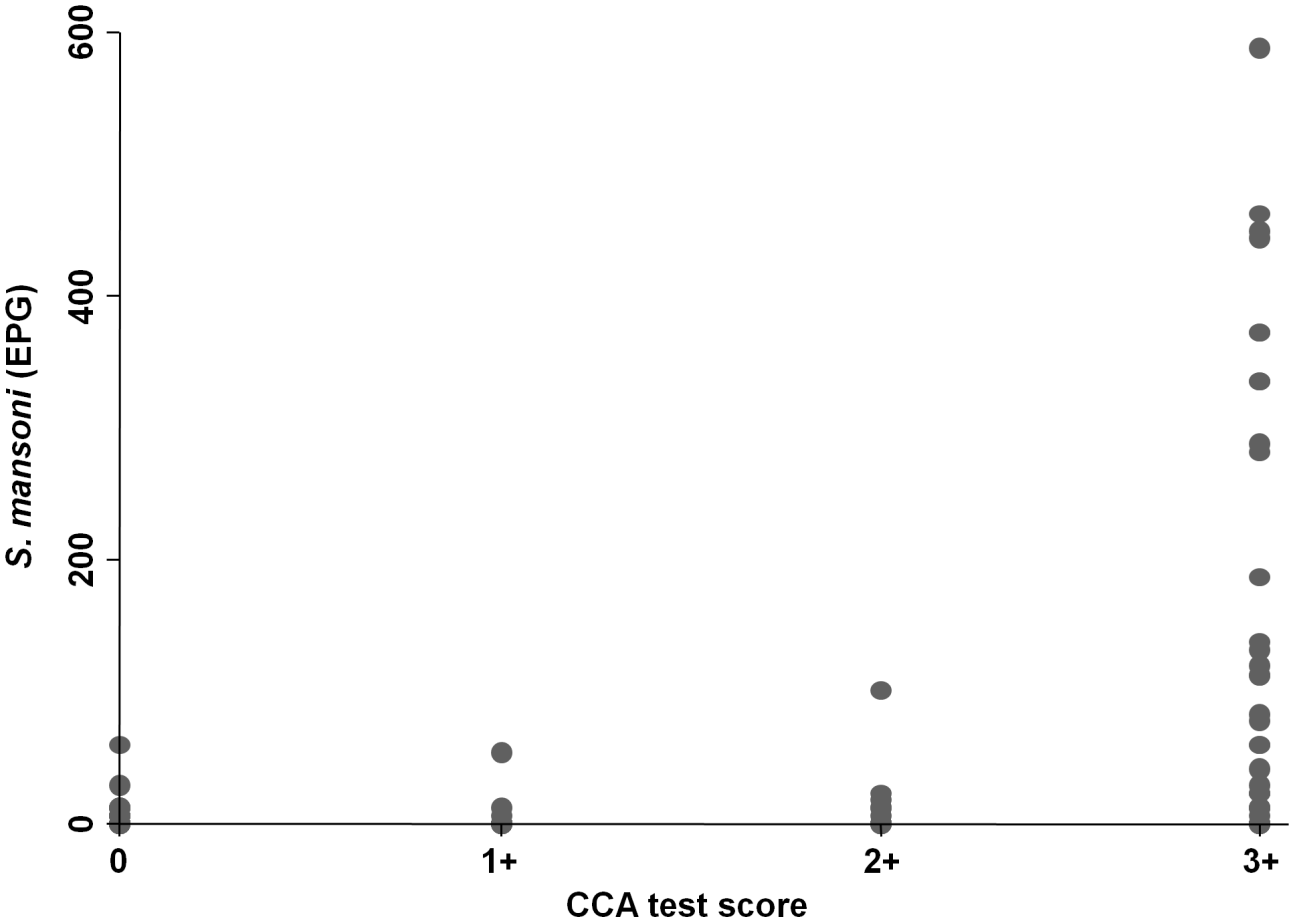
Correlation between *S. mansoni* egg counts and CCA test color reaction scores. This figure shows the correlation between *S. mansoni* eggs per gram of stool (EPG) values, as determined by quadruplicate Kato-Katz thick smears, and a single urine CCA cassette test with ‘trace’ considered as negative result (negative (0), 1+, 2+, and 3+.

Figure 2 shows the correlation between the intensity of *S. mansoni* infection determined by quadruplicate Kato-Katz thick smears, as expressed in EPG, and the CCA(t−) test shown in color scores. We observed a correlation between the color intensity of CCA(t−) test bands and EPG values (odds ratio (OR) = 1.2, p = 0.04).

Comparing the two different methods used for the diagnosis of *S. mansoni*, we found moderate agreement between a single CCA(t−) test and quadruplicate Kato-Katz thick smears (κ = 0.47, p<0.001, Table 3). The agreement between duplicate CCA(t−) and quadruplicate Kato-Katz thick smears was only fair (κ = 0.36, p<0.001). Agreement between the two methods was weaker when considering trace results as positive in the urine CCA cassette test.

**Table 3:** Agreement between Kato-Katz technique and POC-CCA cassette test for the diagnosis of *S. mansoni*.

		Quadruplicate Kato-Katz thick smears		κ*	p
POC-CCA cassette test results	Test result	Positive	Negative		
Single POC-CCA test (t−)	Positive	43	40		
	Negative	13	146	0.47	<0.001
Duplicate POC-CCA tests (t−)	Positive	46	63		
	Negative	10	123	0.36	<0.001
Single POC-CCA test (t+)	Positive	52	104		
	Negative	4	82	0.23	<0.001
Duplicate POC-CCA tests (t+)	Positive	53	132		
	Negative	3	54	0.13	<0.001

The study was carried out in Azaguié, south Côte d'Ivoire in August and September 2011.

* κ indicating kappa; κ<0, no agreement; κ = 0–0.2, poor agreement; κ = 0.21–0.4, fair agreement; κ = 0.41–0.6, moderate agreement; κ = 0.61–0.8, substantial agreement; κ = 0.81–1.0, almost perfect agreement [35].

t−, trace negative; t+, trace positive.

According to our ‘gold’ standard, the sensitivity of a single CCA(t−) test (69.7%) was considerably higher than that of a single (28.3%) or quadruplicate Kato-Katz thick smears (47.5%, Table 4). Also the NPV of a single CCA(t−) test (77.4%) was higher than that of a single (59.1%) or quadruplicate Kato-Katz (65.9%). The sensitivity and NPV of a single CCA(t+) test were higher than those of quadruplicate Kato-Katz and single CCA(t−) (sensitivity: 89.1%; NPV: 84.9%). The specificity of the Kato-Katz technique and CCA(t−) was 100% by definition, whereas the specificity of a single CCA(t+) was considerably lower (59.3%).

**Table 4:** Sensitivity, specificity, and negative predictive value (NPV) of different approaches for the diagnosis of *S. mansoni*.

	Before treatment (*n* = 242)			After treatment (*n* = 86)		
Combined results as ‘gold’ standard*	Sensitivity	Specificity	NPV	Sensitivity	Specificity	NPV
	(95% CI)	(95% CI)	(95% CI)	(95% CI)	(95% CI)	(95% CI)
Single Kato-Katz thick smear	28.3 (20.5–37.3)	100 (97.0–100)	59.1 (52.1–65.9)	4.0 (0.1–20.4)	100 (94.1–100)	71.8 (61.0–81.0)
Duplicate Kato-Katz thick smears	36.1 (27.5–45.4)	100 (97.0–100)	61.8 (54.7–68.6)	4.0 (0.1–20.4)	100 (94.1–100)	71.8 (61.0–81.0)
Triplicate Kato-Katz thick smears	42.9 (33.8–52.3)	100 (97.0–100)	64.4 (57.2–71.2)	4.0 (0.1–20.4)	100 (94.1–100)	71.8 (61.0–81.0)
Quadruplicate Kato-Katz thick smears	47.5 (38.3–56.8)	100 (97.0–100)	65.9 (58.6–72.7)	8.0 (0.9–26.0)	100 (94.1–100)	72.6 (61.8–81.8)
Single POC-CCA cassette test (t−)	69.7 (60.7–77.8)	100 (97.0–100)	77.4 (70.1–83.6)	80.0 (59.3–93.2)	100 (94.1–100)	92.4 (83.2–97.5)
Duplicate POC-CCA cassette test (t−)	91.6 (85.1–95.9)	100 (97.0–100)	92.5 (86.6–96.3)	96.0 (79.6–99.9)	100 (94.1–100)	98.4 (91.3–100)
Single POC-CCA cassette test (t+)	89.1 (81.2–93.5)	59.3 (50.1–68.1)	84.9 (75.5–91.7)	84.0 (63.9–95.5)	77.0 (64.5–86.8)	92.2 (81.1–97.8)
Duplicate POC-CCA cassette test (t+)	97.5 (92.8–99.5)	99.5 (90.1–100)	93.0 (83.0–98.1)	100 (86.3–100)	96.6 (93.1–100)	100 (90.3–100)

The study was carried out in Azaguié, south Côte d'Ivoire in August and September 2011. Sensitivity, specificity, and NPV of different approaches for the diagnosis of *S. mansoni* were assessed before and after praziquantel administration.

* Combined results of quadruplicate Kato-Katz thick smears and duplicate POC-CCA cassette tests with trace results considered as negative.

CI, confidence interval, t−, trace negative; t+, trace positive.

### Diagnostic Accuracy after Treatment

Among the 86 individuals who had complete data records after treatment, *S. mansoni* eggs were detected by Kato-Katz from 22 (25.6%) individuals during the baseline cross-sectional survey. A single POC-CCA, considering trace results as negative, revealed 34 preschoolers (39.5%) with an infection. Considering trace results as positive, then a considerably higher number of preschoolers were classified as positive (*n* = 56, 65.1%).

After treatment, among these 86 children, eggs of *S. mansoni* were only found in two (2.3%) individuals. A single urine CCA(t−) cassette test revealed 20 children (23.3%) with *S. mansoni*, whereas CCA(t+) found 35 (40.7%) infections.

At the 3-week posttreatment evaluation, and considering our ‘gold’ standard (combined results of quadruplicate Kato-Katz thick smears plus duplicate urine CCA(t−) cassette tests), a single CCA(t−) revealed a sensitivity and NPV of 80.0% and 92.4%, respectively (Table 4). Single and even quadruplicate Kato-Katz thick smears showed very low sensitivity (4.0% and 8.0%, respectively) and only moderate NPV (71.8–72.6%).

In our cohort of 86 children, when considering the combined results from both sampling days, 27 children had a positive POC-CCA cassette test result, traces included. Among these children, 20 were *S. mansoni* egg-negative at the baseline survey, whereas the seven infected children had baseline FECs ranging between 6 and 450 EPG. When considering POC-CCA trace results as negative, 24 children were still found with a positive POC-CCA cassette test. Among them, 21 children were egg-negative, whereas the three infected children showed baseline FECs ranging between 132 and 588 EPG. Hence, regardless of whether POC-CCA trace results were considered positive or negative, more than three-quarter of the children found positive with the POC-CCA cassette test at the posttreatment follow-up were egg-negative at the baseline survey.

### Day-to-Day Variability of POC-CCA Cassette Test Scores

Table 5 shows the day-to-day variability of the POC-CCA cassette test scores before (*n* = 242) and 3 weeks after the administration of praziquantel (*n* = 86). At baseline 156 (64.5%) and 145 (59.9%) were found CCA positive on day 1 and day 2, respectively. After treatment, 35 (40.7%) children on day 1 and 32 (37.2%) children on day 2 showed a positive POC-CCA test. Comparing POC-CCA cassette test results from both days, revealed no statistically significant difference in test results before (p = 0.619) and after (p = 0.756) treatment.

**Table 5:** Number of preschool-aged children falling in each POC-CCA test score before and after treatment.

POC-CCA cassette test score	Before treatment (*n* = 242)					After treatment (*n* = 86)				
	Negative (0)	Trace	1+	2+	3+	Negative (0)	Trace	1+	2+	3+
Day 1	86	73	40	14	29	51	15	10	9	1
Day 2	97	65	28	23	29	54	17	9	3	3
Combined scores (days 1 and 2)^a^	105	28	50	27	32	59	3	15	7	2
Higher score (either day 1 or day 2)^b^	57	76	47	23	39	35	31	8	8	4

*n* = 86, Day 1: first day of urine collection, Day 2: second day of urine collection.

a Combined POC-CCA cassette test (days 1 and 2), as defined in Table 1.

b The higher POC-CCA cassette test score from either day 1 or day 2 was considered as final score.

There was relatively little day-to-day variation, both before and after treatment. For example, before treatment, about half of the paired POC-CCA test results showed the same scores, whereas 127 (52.5%) children had discordant scores, with the highest discrepancy observed between negative and trace results. Considering trace results as negative, the percentage of discordant results decreased to 22.7%. In the posttreatment survey, none of the children with duplicate POC-CCA cassette tests performed showed 3+ scores on both days. Discordant POC-CCA test scores between days 1 and 2 were found in slightly more than half of the children (*n* = 44, 51.2%) with the highest number of discordant results between negative and trace results. The concordance between POC-CCA cassette test scores from days 1 and 2 increased with infection intensity (based on POC-CCA cassette test band color), both before and after treatment (Table S1).

**Figure 3:**
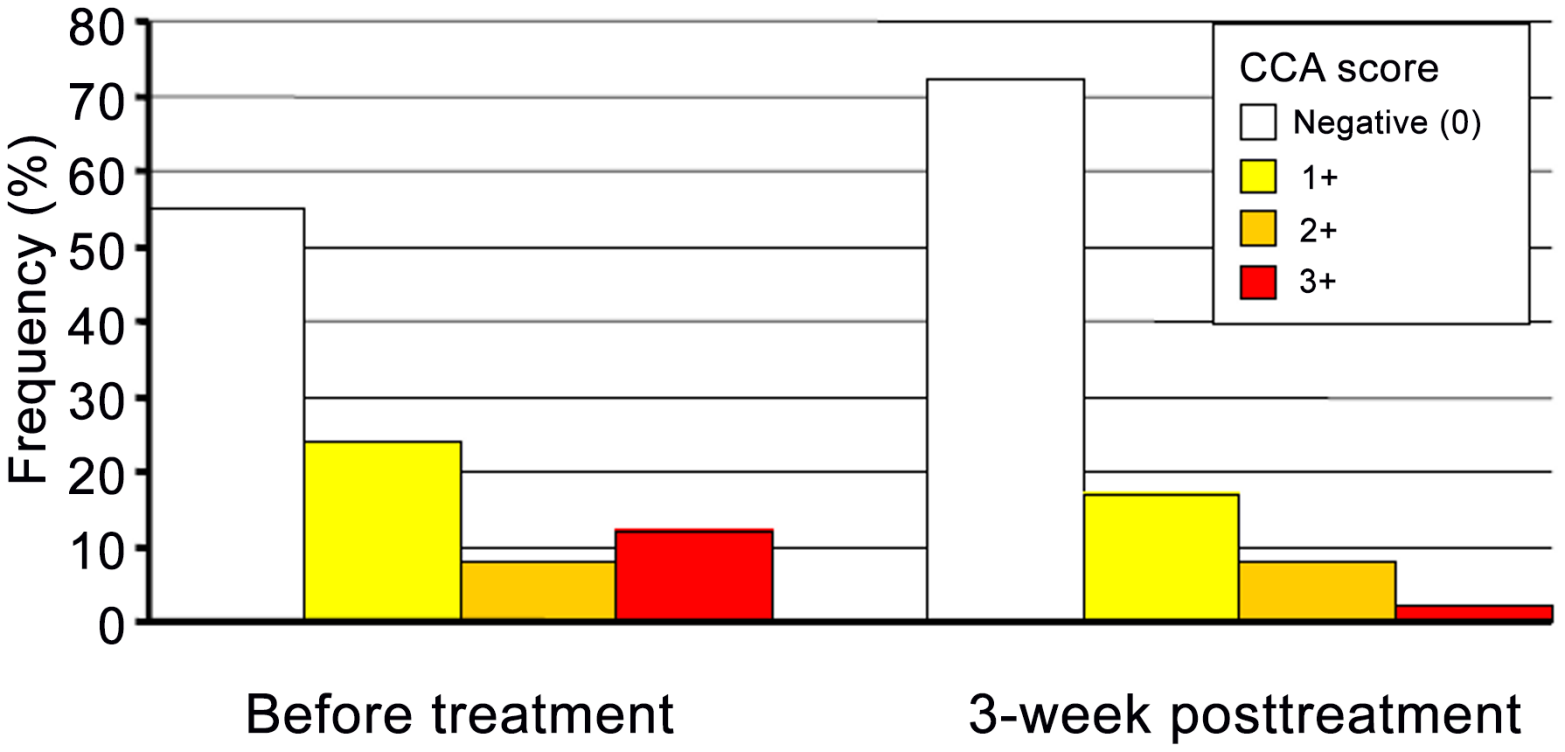
Frequency of CCA test scores before (*n* = 242) and after praziquantel administration (*n* = 86). The frequency of the CCA test scores (0, 1+, 2+, and 3+) before and after treatment with praziquantel was determined based on combined scores from days 1 and 2, as show in Table 1. Note that trace results were considered as negative.

Among those 86 preschool-aged children who had complete data records before and after treatment, and considering the higher of the two color reactions in the duplicate POC-CCA tests as the final score showed that the number of tests scored 3+ before treatment decreased by 76.5% following treatment. A decrease of 22.5% of POC-CCA tests scored as trace was observed 3 weeks posttreatment. Among seven preschool-aged children scored as trace-positive before treatment, four became CCA-negative following treatment, whereas the remaining three were diagnosed CCA-positive (two children with 1+ and one child with 2+). Nine (16.1%) children among the 56 children detected with CCA (trace included) had unchanged test scores after treatment. The number of children found CCA-negative increased sharply 3 weeks after a single dose of praziquantel, with a particularly steep decrease of heavy infections (χ^2^ = 6.50, p = 0.011) (Figure 3).

### Test Requirements of POC-CCA Cassette and Kato-Katz

Table 6 summarizes key test requirements and compares them between Kato-Katz (standard test) and POC-CCA (newly developed test) for the diagnosis of *S. mansoni*. Important test requirements include the ease of obtaining and analyzing the samples, cost considerations, and diagnostic accuracy.

**Table 6:** Comparison of test requirements of POC-CCA cassette test and Kato-Katz technique.

Test requirement	POC-CCA cassette test	Kato-Katz technique
Sample	Urine	Stool
Stage of worm detected	Immature and adult worms through antigens	Adult worms through eggs
Number of sample needed for accurate diagnosis	One sample, even in low endemicity setting	Several samples, especially in low endemicity setting
Sample collection	Straightforward	Difficult, reluctance to provide stool, especially among adults
Time spent to obtain test result at the laboratory	25 min	Several hours
Skill of the person who performs the test	Non-specialized personnel	Specialized personnel
Logistic	Car for transport, POC-CCA test kit	Car for transport, Kato-Katz kit, microscope, microscope slide, electricity

No detailed requirements of each test are mentioned in this table, but the main requirements of each test are emphasized.

## Discussion

There is growing awareness that in high endemicity settings, schistosomiasis already affects preschool-aged children, and hence these young children might need to be included in deworming campaigns [11], [13]–[16]. The Kato-Katz technique has been the backbone of intestinal schistosomiasis (and soil-transmitted helminthiasis) diagnosis in epidemiological studies for decades. However, it shows a low sensitivity for detecting low-intensity infections, which are commonly seen in young children and in communities undergoing regular treatment [21], [28], [36]. Recent studies have shown that a commercially available, urine-based POC-CCA cassette test is a promising method for the diagnosis of *S. mansoni* in preschoolers and school-aged children [13], [23], [26], [28], [29], [37], [38]. In the present work, we investigated the accuracy of this POC-CCA cassette test in preschool-aged children from south Côte d'Ivoire before and after administration of a single oral dose of praziquantel (40 mg/kg) and compared its performance to that of multiple Kato-Katz thick smears.

We found that a single POC-CCA is more sensitive than quadruplicate Kato-Katz thick smears before and 3 weeks after praziquantel treatment. The intensity of a positive CCA test band reaction was significantly correlated with the *S. mansoni* egg burden quantified by the Kato-Katz technique. There was a sharp decrease of CCA tests scored 3+ after treatment and an increase in tests scored negative or trace. The youngest child identified as infected with *S. mansoni* applying the POC-CCA cassette test was 3 months old. Eggs in stool examined with the Kato-Katz method were only detected in children aged 8 months and above.

Our results corroborate recent findings from Kenya and Uganda, where CCA tests detected *S. mansoni* infections in preschool-aged children considerable earlier and at higher frequency than the Kato-Katz technique and an enzyme-linked immunosorbent assay (ELISA) kit to test for host antibodies to soluble egg antigens [13], [28], [29]. The results reported here also extend on our own recent observations in the same study area and that of other groups made elsewhere that urine POC-CCA tests show a considerably higher sensitivity than the widely used Kato-Katz technique for the diagnosis of *S. mansoni* in school-aged children [23], [26], [38]. As confirmed in the present study, the prevalence and intensity of *Schistosoma* infections in preschool-aged children is rather low [9], [11], [13], [31]. Hence, the Kato-Katz and other direct diagnostic methods have limitations when it comes to accurate individual diagnosis. Moreover, the consistency of stools in very young children is mostly diarrheic what renders the preparation of Kato-Katz thick smears difficult, which further challenges an accurate diagnosis. The constrains of using diarrheic stool as well as stool of breastfed infants for helminth diagnosis has been reported elsewhere [39], [40]. In that respect, one needs to consider that in the humid tropics, viral, bacterial, and multiple species parasitic infections causing diarrhea are very common [41]–[43], and that preschool-aged children are particularly prone to such infections [42], [44]. Hence, the Kato-Katz technique has shortcomings for helminth diagnosis in this age-group.

The implementation of large-scale schistosomiasis control programs that are based on preventive chemotherapy reduces the prevalence and, most importantly, the intensity of *Schistosoma* infections [4], [5], [45]. Hence, the endemicity is lowered, which goes hand-in-hand with a reduced accuracy of the Kato-Katz technique [22], [46]. In view of recent discussions regarding schistosomiasis elimination [47], the need for highly sensitive and specific diagnostic tools for the diagnosis of *S. mansoni* and other *Schistosoma* species after extensive preventive chemotherapy campaigns and additional interventions cannot be emphasized enough. However, some weaknesses seem to go against the use of POC-CCA as a diagnostic tool for control programs. First, the Kato-Katz method allows for diagnosis of other helminth infections (e.g., soil-transmitted helminthiasis), which commonly co-exist where schistosomiasis is endemic. Second, the Kato-Katz technique provides a quantitative measure to the infections, which guide the control program interventions. Third, the cost of a single POC-CCA cassette (approximately US$ 1.75) is similar to the total costs of performing a single Kato-Katz thick smear in epidemiological surveys (US$ 1.7) [48], [49]. Hence, the costs for individual diagnosis currently limit the use and attractiveness for program managers for larger-scale applications. For individual diagnosis, however, it should be noted that the costs largely depend on the patient's economical situation.

Our finding of very young children diagnosed with *S. mansoni* when using the urine POC-CCA cassette test (3 months old), and only 5 months later when using the Kato-Katz technique raises an alarm bell. Current control programs focus on the school-aged population (usually starting at an age of 5–6 years), and hence a considerable number of infected children might be restrained from treatment for perhaps 3–4 years. Recent studies discussed the potential impact of early infections that remain untreated for several years on child health due to the cumulative effect of repeated infections [23], [50]–[52]. Our observations are also important from a surveillance point of view. Indeed, first the POC-CCA test revealed the age of first *S. mansoni* infection several months earlier than the Kato-Katz technique and, second, we found that three-quarter of the people who were CCA-positive at follow-up were egg-negative at baseline. It seems that these children were infected with immature worms that praziquantel was not able to kill. Hence, despite the aforementioned limits of the POC-CCA cassette test, some advantages deserve to be highlighted. First, POC-CCA is based on simple-to-use urine test, which can be performed by non-specialized personnel. Hence, it can be employed in remote rural areas that lack access to the power grid by minimally trained people (Table 6). Second, collection of urine samples for POC-CCA is more straightforward and less invasive than collection of stool for Kato-Katz thick smears. The time spent from the field (sample collection; urine for POC-CCA cassette test *versus* stool for Kato-Katz thick smears) to the laboratory (implementation; at least 25 min for POC-CCA cassette test *versus* several hours for Kato-Katz thick smears) places the POC-CCA in a favorable position. Third, a POC-CCA test is able to detect prepatent infections, whereas the Kato-Katz technique can only detect patent infections. Note that de Water and colleagues, in the mid-1980s, studying ultrastructural localization of CCA in the digestive tract of various life-cycle stages of *S. mansoni* showed that the antigens are present in the gut of adult worms, as well as in the primordial gut cells of cercariae aged 3.5 weeks [53]. In addition, a study implemented by van Dam and colleagues 10 years later on *in vitro* and *in vivo* excretion of CAA and CCA by developing schistosomula and adult worms showed that during the first days of *S. mansoni* development more CAA than CCA was excreted, while after one week the trend was reversed [25]. Taken together, the POC-CCA cassette test is an adequate and most useful tool for rapid identification of infected individuals and high-risk communities that warrant interventions at the individual patient level and at the community level with the goal to lower morbidity and transmission of schistosomiasis. Efforts might thus be warranted by the United Nations through its agencies to allow extension of the use of POC-CCA tests in schistosome-endemic areas where financial resources are often limited.

Our study shows that the number of positives determined by POC-CCA after treatment is considerably higher than that revealed by quadruplicate Kato-Katz thick smears. Indeed, the Kato-Katz technique found a very low prevalence after treatment (2.3%), whereas POC-CCA test results revealed several-fold higher prevalences (23.3% considering trace results as negative and 40.7% considering trace results as positive). These differences might be explained by the following reasons. First, the Kato-Katz technique is underestimating the prevalence due to very low infection intensities after treatment [54]. Second, in the current study, Kato-Katz thick smears were read shortly after slide preparation (within 30–60 min). Prompt microscopic examination of Kato-Katz thick smears is recommended for the concurrent diagnosis of soil-transmitted helminths, particularly hookworm [20], but the optimal detection of *S. mansoni* eggs is after clearing the slides for 24 hours [55]. On the other hand, the POC-CCA test might overestimate the prevalence (i.e., in case CCA is still excreted in urine more than 3 weeks after treatment despite the death of adult worms). Studies conducted to date are inconclusive on when exactly CCA is eliminated from urine below detection limits [25], [56], [57]. In view of the aforementioned limitations of our direct parasitological approach, it is conceivable that CCA-positive, egg-negative cases are false-negatives based on the Kato-Katz technique [33].

Assessing the converting proportion of POC-CCA test color band scores after treatment, we observed a significant increase of negative scores and decrease of trace and 3+ scores, despite only considering the combined score per individual over two test days. Since we also found that the FECs detected with the Kato-Katz method correlate with the test band color intensity, the POC-CCA test might indeed reveal formerly heavily infected individuals as still positive. In light of the absence of a real ‘gold’ standard in our study, future investigations using highly sensitive and specific diagnostic methods (i.e., a polymerase chain reaction (PCR) [58], or detection of CAA by an up-converting phosphor technology (UPT)-based lateral flow (LF) assay [59]) are of need to investigate the true accuracy of a urine CCA cassette test after treatment, and hence its applicability for monitoring the success of schistosomiasis control programs.

In conclusion, a single POC-CCA urine cassette test appears to be more sensitive than multiple Kato-Katz thick smears for the diagnosis of *S. mansoni* in preschool-aged children. It is therefore an appropriate tool for the rapid identification of *S. mansoni*-infected individuals, including preschool-aged children, and of high-risk communities before the onset of control interventions. Its applicability to accurately assess infections a few weeks after praziquantel administration needs further investigation and comparison with highly sensitive and specific diagnostic tools.

## Supporting Information

Alternative Language Abstract S1 **Translation of the Abstract into French by Jean T. Coulibaly.**
(DOC)

Checklist S1 **STARD Checklist.**
(PDF)

Table S1 **Concordance between POC-CCA test scores from consecutive days 1 and 2 at individual level before and after treatment.**
(DOC)
