# Supplementary material for: Accuracy of Urine Circulating Cathodic Antigen Test for the Diagnosis of Schistosoma mansoni in Preschool-Aged Children before and after Treatment
Source: PLoS Negl Trop Dis. 2013 Mar 21;7(3):e2109. doi: 10.1371/journal.pntd.0002109 (PMC3605147; doi:10.1371/journal.pntd.0002109)
Supplement: Alternative Language Abstract S1 — Translation of the Abstract into French by Jean T. Coulibaly. (DOC) [file pntd.0002109.s001.doc]

**Fiabilité d’un Test Basé sur les Antigènes Cathodiques Circulants de l’Urine pour le Diagnostic de *Schistosoma mansoni* chez les Enfants d’Âge Préscolaire Avant et Après Traitement**

**Résumé**

***Contexte:*** La technique de Kato-Katz est largement utilisée pour le diagnostic de *Schistosoma mansoni*, mais montre une faible sensibilité chez les individus dont l’intensité de l’infection est légère. Nous avons évalué la fiabilité d’un test de diagnostic rapide, disponible dans le commerce, qui est basé sur la détection d’antigènes cathodiques circulants (POC-CCA) pour le diagnostic de *S. mansoni* chez les enfants d’âge préscolaire avant et après le traitement de Biltricide.

***Méthodologie:*** Une enquête longitudinale de 3 semaines couplée d’une intervention thérapeutique a été réalisée à Azaguié au sud de la Côte d’Ivoire. Dans l’ensemble, 242 enfants d’âge préscolaire (tranche d’âge: 2 mois à 5,5 ans) ont présenté chacun, deux échantillons de selles et d’urine avant le traitement avec le Biltricide, et 86 de ces enfants ont été suivis après traitement. Des échantillons de selles ont été examinés avec deux Kato-Katz pour le diagnostic de *S. mansoni*. Les échantillons d’urine ont été soumis au test POC-CCA pour le diagnostic de *S. mansoni*, et une méthode de filtration pour le diagnostic de *S. haematobium*.

***Résultats Principaux****:* Avant le traitement, la prévalence de *S. mansoni*, tel que déterminée par quadruple Kato-Katz, un seul test POC-CCA avec les « trace » considéré comme négatif (t-), et un seul test POC-CCA avec les « trace » considéré comme positif (t+), était de 23,1 %, 34,3 % et 64,5 %, respectivement. En utilisant les résultats combinés (soit de quatre Kato-Katz et deux POC-CCA(t-)) en tant que référence, la sensibilité d’une seule lame de Kato-Katz, d’un seul test POC-CCA(t-) ou POC-CCA(t+) était de 28,3 %, 69,7 % et 89,1 %, respectivement. Trois semaines après le traitement, la sensibilité d’une seule lame de Kato-Katz, d’un seul test POC-CCA(t-) et un seul test POC-CCA(t+) était de 4,0 %, 80,0 % et 84,0 %, respectivement. L’intensité de la réaction de la bande du test POC-CCA était corrélée avec la charge en œuf de *S. mansoni* (« odds ratio » = 1,2, p = 0,04).

***Conclusions/Signification:*** Un seul test POC-CCA semble être plus sensible que plusieurs lames de Kato-Katz pour le diagnostic de *S. mansoni* chez les enfants d’âge préscolaire avant et après le traitement de Biltricide. Le test POC-CCA peut être recommandé pour l’identification rapide des infections à *S. mansoni* avant le traitement. Des études supplémentaires sont nécessaires afin de déterminer l’utilité du test POC-CCA pour l’évaluation de l’efficacité des médicaments et le suivi de l’impact des interventions.

Traducteur: Jean T. Coulibaly
