# Supplementary material for: Accuracy of Urine Circulating Cathodic Antigen Test for the Diagnosis of Schistosoma mansoni in Preschool-Aged Children before and after Treatment
Source: PLoS Negl Trop Dis. 2013 Mar 21;7(3):e2109. doi: 10.1371/journal.pntd.0002109 (PMC3605147; doi:10.1371/journal.pntd.0002109)
Supplement: Table S1 — Concordance between POC-CCA test scores from consecutive days 1 and 2 at individual level before and after treatment. (DOC) [file pntd.0002109.s003.doc]

**Table S1 Concordance between POC-CCA test scores from consecutive days 1 and 2 at individual level before and after treatment.**

|  | **Before treatment (n = 242)** | | | | | **After treatment (n = 86)** | | | | |
| --- | --- | --- | --- | --- | --- | --- | --- | --- | --- | --- |
|  | **Day 2** | | | | | | | | | |
| **Day 1** |  |  |  |  |  |  |  |  |  |  |
| **Urine CCA cassette score** | **Negative (0)** | **Trace** | **1+** | **2+** | **3+** | **Negative (0)** | **Trace** | **1+** | **2+** | **3+** |
| **Negative (0)** | 57 | 20 | 5 | 3 | 1 | 36 | 11 | 2 | 2 | 0 |
| **Trace** | 28 | 28 | 12 | 4 | 1 | 13 | 2 | 0 | 0 | 0 |
| **1+** | 9 | 13 | 8 | 7 | 3 | 3 | 3 | 3 | 0 | 1 |
| **2+** | 2 | 3 | 1 | 3 | 5 | 1 | 1 | 3 | 2 | 2 |
| **3+** | 1 | 1 | 2 | 6 | 19 | 1 | 0 | 0 | 0 | 0 |
